# Grooved vs smooth ureteric stent before extracorporeal shockwave lithotripsy: Single-blind randomised clinical trial

**DOI:** 10.1080/2090598X.2021.2004502

**Published:** 2021-12-07

**Authors:** Abdulqadir Alobaidy, Tarek Ibrahim, Walid El Ansari, Hosam Tawfik, Abdulla Al-Naimi, Salam Hussain, Abdulla Al-Ansari

**Affiliations:** a Department of Surgery, Urology Section, Hamad Medical Corporation, Doha, Qatar; b Department of Surgery, Hamad General Hospital, Hamad Medical Corporation, Doha, Qatar; c College of Medicine, Qatar University, Doha, Qatar; d School of Health and Education, University of Skovde, Skovde, Sweden

**Keywords:** Grooved stent, lithotripsy, randomised controlled trial, stone, ureteric stent

## Abstract

**Objective:**

No study compared the grooved stent to the widely used standard smooth (non-grooved) stent in humans. We compared stone clearance, complications, and patient tolerance of the grooved stent vs standard JJ stent.

**Patients and Methods:**

Single-blinded randomised trial among patients planned for pre-extracorporeal shockwave lithotripsy (ESWL) stenting. Adult patients with unilateral ureteric/renal stones planned for ESWL were randomly assigned to receive (Percuflex) smooth ureteric stent or (Visiostar) grooved lithotripsy stent and blinded to the stent type. We collected and compared the baseline data and outcomes (stone-free rate, complications, and stent-related symptoms) of both patient groups.

**Results:**

A total of 96 adults were included (48 per arm). There were no significant differences between the groups at baseline in terms of demographics, body mass index, comorbidities, renal function, number of ESWL sessions, and stone characteristics, including pre-ESWL stone volume (mean [SD] smooth 310.2 [301.6] vs grooved 270.7 [278.6] mm3, P = 0.5). Stone clearance was statistically insignificant between the groups, although clinically relevant (smooth stent 70.8% vs grooved stent 81.2%, P = 0.2). Grooved-stent patients reported comparable urinary symptoms score (*P =* 0.05) and operative complications (*P =* 0.6), but significantly more urinary tract infections (UTIs) not requiring hospitalisation (*P =* 0.003).

**Conclusions:**

Although statistically insignificant, the grooved stent exhibited higher stone clearance compared to the smooth stent, with similar complication rates excpet that patients with grooved stents reported more UTIs. A re-visit to the size of the outer diameter of the grooved stent could enhance its stone clearance properties, and further development of its coating material could lead to better patient satisfaction.

## Introduction

Urolithiasis is a common disease with increasing prevalence especially in the Middle East [1]. Despite the technological advances in surgical management, extracorporeal shockwave lithotripsy (ESWL) remains a viable treatment option for renal and proximal ureteric stones [2]. Due to its low invasiveness, cost, and complications as well as high efficiency, ESWL provides a stone-free rate (SFR) of up to 95% [3–5]. The American Ureteral Stones Guidelines panel recommended ESWL as the first-line treatment for ureteric stones of ≤1 cm and optional for those >1 cm [6]. Likewise, the European and Asian Associations of Urology recommend ESWL as the first option in the treatment of renal stones of <2 cm [2,7]. However, despite its popularity, ESWL has limitations that might discourage some surgeons from the procedure, preferring to use other treatment modalities.

One possible limitation of ESWL for urinary tract calculi is the development of Steinstrasse (‘stone street’), described as a column of stone fragments that forms and blocks the ureter [8]. This complication occurs in 4–7% of patients undergoing ESWL, 46% of whom may require an invasive procedure to relieve the pain and obstruction. Evaluating the risk factors of developing Steinstrasse, some reports found a higher risk with stones of >2 cm, while others reported the complication with smaller stones >1 cm [9,10]. Although ureteric stenting before ESWL can prevent Steinstrasse, findings remain inconsistent [11,12]. Whilst pre-stenting can reduce steinstrasse and subsequent hospitalisation; pre-stenting is associated with disadvantages, e.g. inferior SFR, increased stent-related local irritation, encrustation and infection, and stent-associated events such as migration, breakage and missed stent [5,13,14]. Hence, currently, pre-stenting is performed only in selected patients [14].

In an attempt to avoid such disadvantages, a grooved stent was manufactured to facilitate the passage of stone fragments and in the meantime maintain the advantage of preventing Steinstrasse formation. The star-shaped grooved design of this stent provides room for the easy passage of urine and stones within the grooves [15]. In addition, its aliphatic polyurethane material is alleged to be resistant to encrustation and causes less irritation [15].

The grooved stent was evaluated only once in an animal model [16]. Surprisingly, to the best of our knowledge, there are no clinical studies of grooved stents in humans. Therefore, the present single-blinded randomised controlled trial (RCT) compared the grooved stent to the widely used standard smooth stent. The specific objectives were to compare for both stents, their stone clearance (SFR and duration of clearance), range of complications, and patient satisfaction (urinary symptom/s, body pain, general health, work performance, sexual matters). This is the first study to undertake such comparisons in humans.

## Patients and Methods

### Study design, ethics, and patient selection

This randomised controlled single-blind study was approved and monitored by the Medical Research Center (IRB) of Hamad Medical Corporation, Doha, Qatar (approval #16013/16). All patients admitted to the Department of Urology at Hamad General hospital (largest tertiary care hospital in Qatar) for stenting and planned for SWL were screened for recruitment. The inclusion criteria were adults aged >18 years with unilateral radio-opaque ureteric stone/s and stone burden of >65 mm^3^ who presented to the emergency department complaining of renal colic requiring ureteric stenting and planned for ESWL. The exclusion criteria included radiolucent and bilateral stones, patients planned for ureteroscopy rather than ESWL as a definitive stone management, and in line with the European Association of Urology (EAU) guidelines, patients with any contraindication/s for ESWL, e.g. pregnancy, coagulopathy or use of platelet aggregation inhibitors, aortic aneurysms, severe untreated hypertension, severe skeletal malformations, severe obesity, and anatomical obstruction distal to the stone [2].

**Figure 1.**
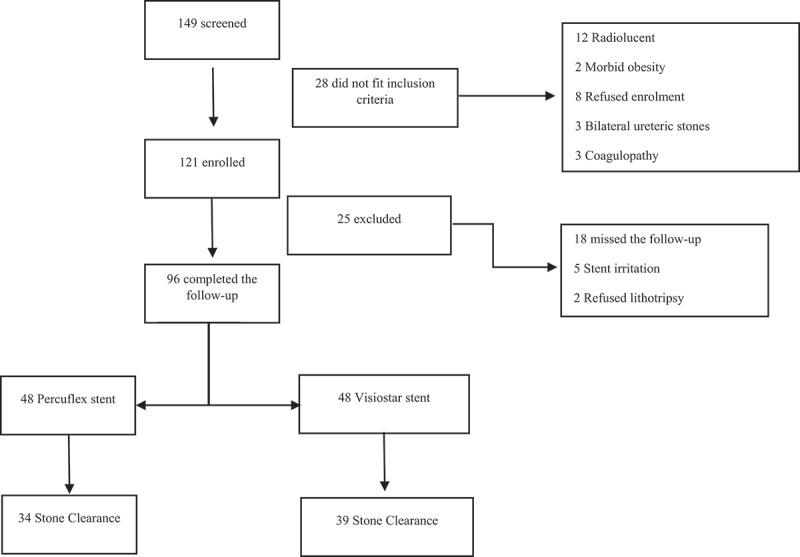
Study CONSORT diagram.

A total of 149 patients underwent ureteric stenting during the study period (June 2018 to September 2019), of whom eight refused enrolment and another 20 did not fit the inclusion criteria and were hence excluded (12 had radiolucent stones, three had bilateral ureteral stones, and three had coagulopathy (Figure 1)). The aims and objectives of the study were explained to the remaining 121 eligible patients, and they were invited to participate in the study. Upon agreement, the patients completed a written informed consent and were enrolled. Of the 121 enrolled patients, 25 did not complete the study and were thus excluded (18 missed scheduled follow-up/s and seven changed their minds [refused lithotripsy and hence were offered and underwent ureteroscopy instead]). The remaining 96 patients who completed the study are included in the present analysis (48 smooth stents, 48 grooved stents), of which 15 did not complete the ureteric stent symptom questionnaire (10 patients from the smooth stent group, and five from the grooved stent group) and hence were not included in the analysis of the questionnaire. Figure 1 depicts the study Consolidated Standards of Reporting Trials (CONSORT) diagram. Good clinical practice guidelines were followed in line with the declaration of Helsinki, identifiable patient data were coded and kept securely stored by the principal investigator, and privacy and confidentiality were observed. Data collected included patients’ age and gender, as well as the findings of renal function tests and stone parameters obtained on low dose non-contrast CT scan.

### Randomisation

**Figure 2.**
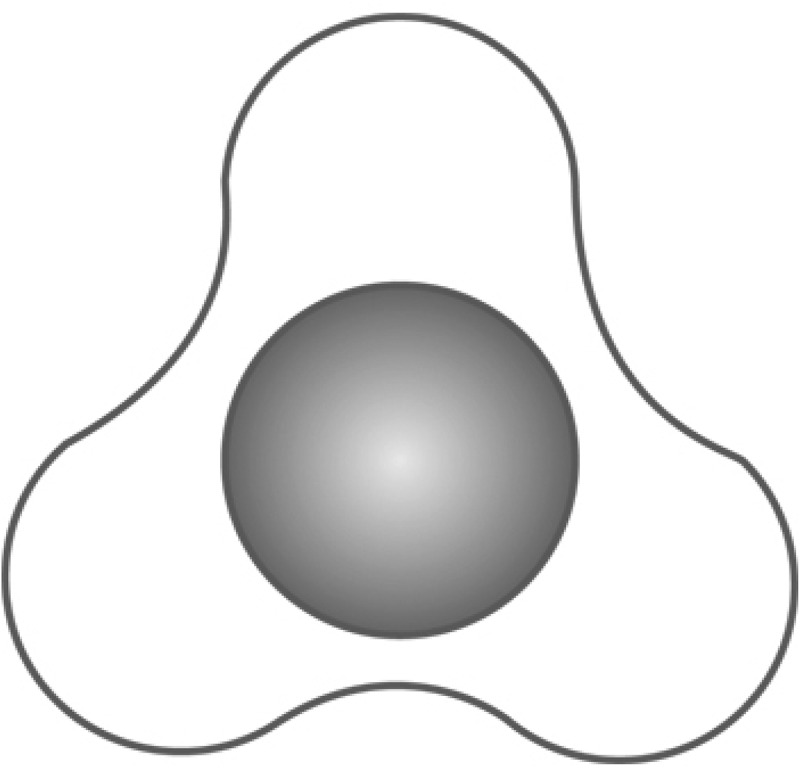
Visiostar (grooved) ESWL stent.

**Figure 3.**
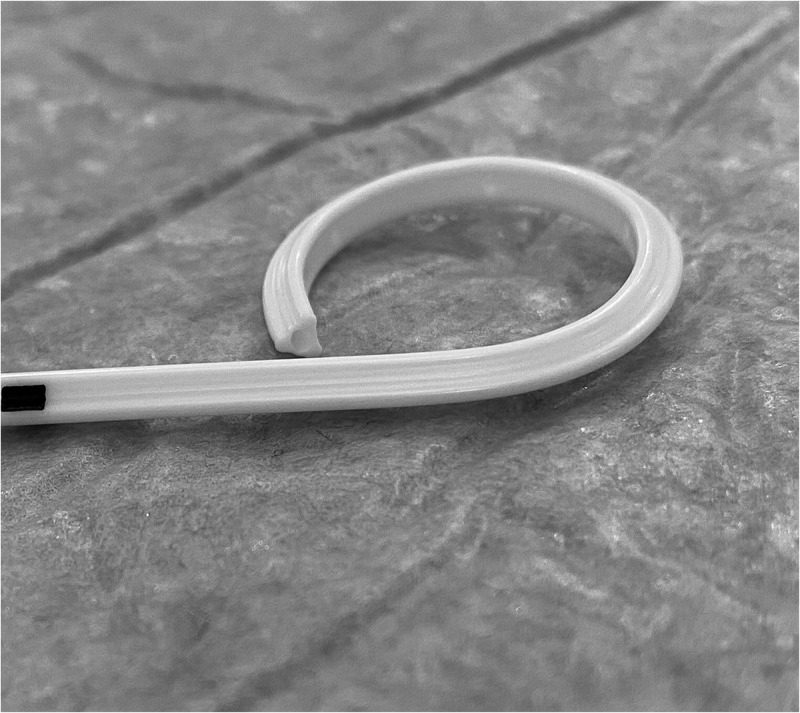
Distal coil of Visiostar lithotripsy stent.

**Figure 4.**
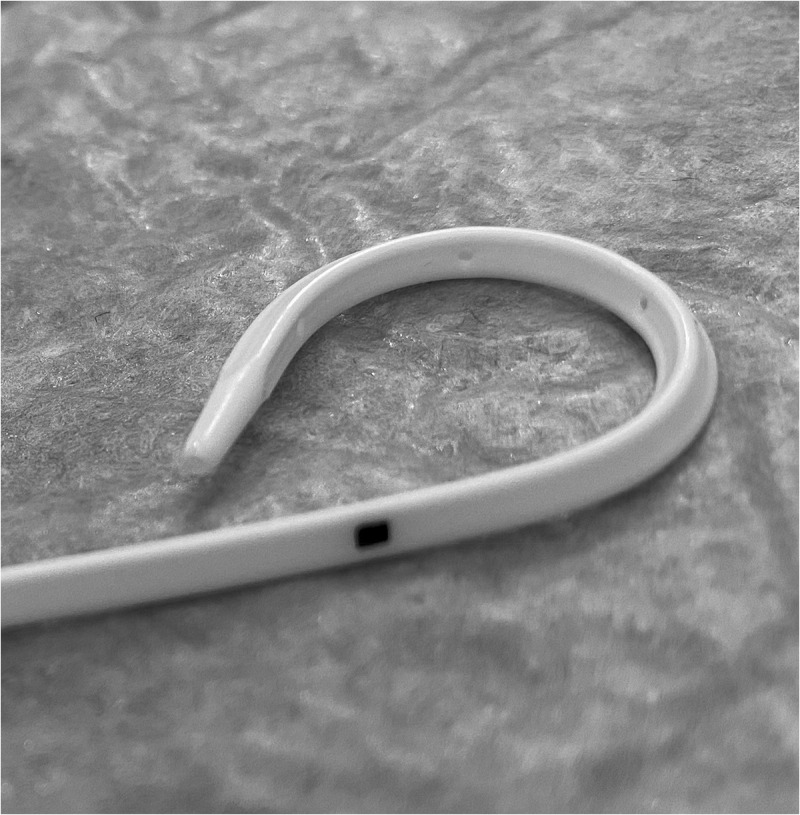
Renal coil of Visiostar lithotripsy stent.

Patients were randomised into two groups according to the stent type. Group A had smooth (Percuflex Plus) 6 F/26-cm stent inserted (Boston Scientific, Marlborough, MA, USA); Group B had grooved (Visiostar-ESWL) 7 F/26-cm stent inserted (Urovision.Urotech, Rohrdorf, Germany) (Figures 2-4). The Visiostar grooved stent is only manufactured in 7 F and thus we had to compare the commercially available grooved stent to the commonly used 6 F smooth stent. Randomisation was computer-generated, and recruited patients were allocated in blocks (two and four) random process. One certified urologist trained by the research team was responsible for the initial recruitment of patients, whilst another trained urologist was responsible for receiving the opaque concealed allocation envelope, opening it, and allocating the patient to either group, revealing the stent type only to the operating surgeon, whilst keeping it concealed from all other members of the research team. This person also had a separate securely-kept sheet that coded each patient to the stent type. Recruited patients were not informed about the group allocation (blinded).

### Surgical procedure

Ureteric stenting was performed by certified urologists under either general or spinal anaesthesia. In the case of UTI, a broad-spectrum antibiotic carbapenem (ertapenem) was started preoperatively or prescribed based on urine culture findings. Prophylactic antibiotics third-generation cephalosporin (ceftriaxone) was given on induction when there was no pyuria or evidence of UTI. Positioning the patients in lithotomy, the procedure starts with a cystoscopic evaluation of the urethra, urinary bladder and ureteric orifices followed by retrograde pyelography evaluation of the upper tract and stone. A guidewire is inserted and then the backloading of the stent over the guidewire is undertakenunder fluoroscopic guidance that confirms the stent is in position.

ESWL was performed ~2 weeks after stenting for a maximum of three sessions, 2 weeks apart, and assessment of stone clearance prior to each session was undertaken using X-ray. ESWL was conducted under fluoroscopic guidance and cardiac monitoring in a supine position and the lithotripsy procedure followed the European guidelines of best practice including the setup, coupling, and analgesia [2]. An electromagnetic machine (M3 Liskope Siemens) was used with a routine setup of 90 shocks/min and low power (0.1 J). The power was gradually ramped up reaching a maximum of 6.0 J. A total of 3000 shocks was used in renal stones, while ureteric stones received 4000 shocks. The stone location and clearance were monitored throughout the procedure by a certified urologist using fluoroscopy in two planes (anteroposterior and oblique).

### Definitions

Before ESWL, stone size was calculated manually as a volume using the ellipsoid algebra formula: a × b × c × π/6 (mm^3^) for accurate assessment of stone burden rather than the maximum dimension [17,18]. 19,20,20,19,20

### Procedures

The same urologist responsible for the initial recruitment of patients assessed the outcomes after ESWL. This person, although blinded to the group allocation could identify the stent type during the X-ray assessment (hence the study is single blinded). We compared the stent groups for stone clearance after the last ESWL session using X-ray, or after ureteroscopy in case of suspected residual stones (for both groups). [20.All patients were prescribed tamsulosin routinely upon insertion of stents.

### Statistical analysis

This is the first RCT to assess the SFR in patients undertaking ESWL in the presence of an implanted grooved or smooth ureteric stent. Hence, formal sample size calculation was not undertaken. A total sample size of 72 patients was deemed appropriate. We further increased the sample size to 121 in order to accommodate any further possible dropouts. Baseline patients’ characteristics between the two stent groups were compared using a *t*-test for quantitative data and a chi-square test for qualitative variables. Intention-to-treat principle was applied on two patients for whom a Visiostar (grooved) stent could not be inserted. The primary outcome variable was SFR assessment in the two intervention groups and was compared using the unpaired *t*-test. Correlation analysis (Pearson’s) was used to examine the relationship between two quantitative variables. The Statistical Package for the Social Sciences (SPSS®) version 20.0 (IBM Corp., Armonk, NY, USA) was used to analyse the data, and *P* < 0.05 was considered statistically significant.

## Results

**Table 1.** Baseline characteristics of the sample

Variable	Stent		*P*
	Smooth (Percuflex) *N* = 48	Grooved (Visiostar) *N* = 48	
Gender, *n* (%)			0.09
Male	47 (97.9)	43 (89.6)	
Female	1 (2.1)	5 (10.4)	
Age, years, mean (SD)	35.1 (9.6)	38.1 (10.9)	0.15
Weight, kg, mean (SD)	72.9 (12.3)	72.15 (11.3)	0.8
Height, cm, mean (SD)	168.5 (6.2)	163.6 (22.5)	0.15
BMI, kg/m^2^, mean (SD)	25.5 (4.1)	26.37 (4.5)	0.3
Comorbidities, *n* (%)			
None	47 (97.9)	42 (87.5)	0.1
Diabetes mellitus	1 (2.1)	5 (10.4)	0.1
Hypertension	1 (2.1)	3 (6.2)	0.3

BMI: body mass index.

**Table 2.** Baseline renal and stone characteristics of the sample

Variable	Stent		*P*
	Smooth (Percuflex) *N* = 48	Grooved (Visiostar) *N*= 48	
Leucocytosis, × 10^6^, mean (SD)	10.9 (3.8)	10.19 (3.2)	0.3
Median	10	9.7	
Creatinine, μmol/L, mean (SD)	109 (31)	103 (32)	0.3
Median	101	98	
Indications, *n* (%)			0.8
Obstruction	23 (47.9)	20 (41.7)	
Pain	16 (33.3)	18 (37.5)	
UTI	9 (18.8)	10 (20.8)	
Stone, *n* (%)			
Laterality			0.4
Right	27 (56.3)	31 (64.6)	
Left	21 (43.8)	17 (35.4)	
Site (after stenting)			0.9
Kidney			
Middle calyx	2 (4.2)	2 (4.2)	
Lower calyx	10 (20.8)	8 (16.7)	
Ureter			
Upper	33 (68.8)	35 (72.9)	
Mid	3 (6.2)	3 (6.2)	
Size, mm^3^, mean (SD)	310.2 (301.6)	270.7 (278.6)	0.5
Median	199.8	182.3	
Stone density, HU, mean (SD)	1128 (317)	1109 (238)	0.7
Median	1084	1049	
SWL, *n* (%)			
Number of sessions			0.4
1	20 (41.7)	26 (54.2)	
2	16 (33.3)	14 (29.1)	
3	12 (25)	8 (16.7)	

HU: Hounsfield units; italics indicate statistical significance

### Baseline demographic characteristics

Table 1 shows the baseline demographic characteristics of the sample. There were no differences in the demography of the two stent groups. Across the whole sample, 89 patients did not have any comorbidities, while the remaining 11 had either diabetes mellitus or hypertension or both.

### Baseline renal and stone characteristics

None of the recruited patients had a significant reduction of renal size or parenchymal thickness of the ipsilateral kidney compared to the other kidney and none had compensatory renal hypertrophy of the contralateral kidney. All the patients had ureteric stones that were indicated for stenting. Of the 96 patients, 77 patients had upper ureteric stones close to the ureteropelvic junction causing intolerable pain, of whom 43 were associated with severe hydronephrosis and impairment of renal function, and 19 patients presented with UTI drained by insertion of ureteric stents. Upon stent insertion, 22 stones migrated to the kidneys. The indications of stenting and the stone location during ESWL in each group are shown in Table 2. Table 2 also shows that there were no statistically significant differences in the baseline renal and stone characteristics of both stent groups.

### Comparison of outcomes by stent type

Table 3 compares the stone clearance and complications between stent types. The Visiostar grooved stent had a statistically insignificantly higher SFR within a shorter duration of clearance (grooved stent 81.2% vs smooth stent 70.8%, *P* = 0.2). The overall stone clearance rate across both stent groups was 76%. The complication rate was comparable for both stent types. The Visiostar grooved stent could not be inserted in two patients and hence a Percuflex smooth stent was inserted instead. For both these patients, the operating urologists reported feeling resistance at the stone site while inserting the Visiostar stent, while such resistance was not felt during insertion of the Percuflex stent for the same patients. One of these two patients was cleared by ESWL, while the other required auxiliary ureteroscopy. Contrast extravasation during stenting occurred in two patients (one patient from each group). All complications were classified Grade I as per the Clavien–Dindo grading system.

**Table 3.** Comparison of outcomes among the Percuflex and Visiostar stent groups

Variable	Stent		*P*
	Smooth (Percuflex) *N* = 48	Grooved (Visiostar) *N* = 48	
Outcome, *n* (%)			
Stone clearance	34 (70.8)	39 (81.2)	0.2
Stenting operative complications	1 (2.1)	3 (6.2)	0.6
ESWL complications	0 (0)	0 (0)	0.3
Stent duration, days, mean (SD)	29 (18.6)	26.5 (15.3)	0.5

### Patient satisfaction

The USSQ assessed the stent-related patient satisfaction. Table 4 shows the comparison of stent tolerance among patients that received the Percuflex smooth and Visiostar grooved stents. In terms of the irritation caused by the stents, patients with Percuflex stents reported less urinary symptoms (*P* = 0.05), less UTIs/less antibiotic usage (*P* = 0.003). As for the remaining USSQ domains, there were no significant differences for body pain, general health, work performance, and sexual matters across both patient groups.

**Table 4.** Comparison of USSQ outcomes among patients with Percuflex and Visiostar stents

USSQ domains	Stent		*P*
	Smooth (Percuflex) *N* = 38	Grooved (Visiostar) *N* = 43	
Mean (SD):			
Urinary symptom/s	26.7 (4.4)	29.1 (6.4)	0.05
Body pain	16.8 (5.1)	15.5 (7.2)	0.3
General health	15.2 (3.25)	15.5 (3.75)	0.7
Work performance	5.6 (2.25)	5.63 (2.4)	0.9
Sexual matters	4.6 (1.8)	5.1 (2.2)	0.2
UTI/antibiotics	3.8 (1.7)	5.5 (2.9)	*0.003*
General satisfaction	1.1 (0.3)	1.4 (0.7)	0.06

Italics indicates statistical significance. The USSQ was completed 5–6 weeks after the placement of the ureteric stent.

## Discussion

ESWL is considered the least invasive intervention for urolithiasis. Stenting before ESWL may be needed in selected cases to prevent Steinstrasse after lithotripsy of large stones, to drain obstructive ureteric stones that cause infection, or to dilate a tight ureter subsequent to failed ureteroscopy [2]. However, stenting has drawbacks. First, pre-stenting might affect the ESWL SFR. Older studies [21,22] found no reduction in the SFR in pre-stented patients, but recent evidence observed an 83.7–95% SFR in unstented patients vs 68.6–84.9% in stented [5,23,24]. In addition, stent placement could result in unsatisfactory symptoms and complications [13,22].

Most commercially available stents are smooth walled with a rounded circumference, hence most research has assessed such stents [12–14,20–24]. Comparison of smooth stent to the grooved stent has been undertaken only in animals [16]. To date, no study had assessed the use of the more recently designed grooved stent or compared its use to the smooth stent among humans.

Therefore, the present RCT compared the most commonly used Percuflex smooth stent to the Visiostar grooved stent in terms of stone-clearance rates, complications, and patient satisfaction. Our main findings were that the Visiostar grooved stent had a statistically insignificant better stone clearance, no differences in operative complications (*P* = 0.6), except that Visiostar patients reported in the USSQ significantly more UTIs (*P* = 0.003) that were managed by empiricaloral antibiotics with no need for admission. There were no differences between both stents in the satisfaction domains of body pain (*P* = 0.3), general health (*P* = 0.7), work performance (*P* = 0.9) and sexual matters (*P* = 0.2) as measured by the USSQ, although the Visiostar stents caused more urinary symptoms that were borderline significant (*P* = 0.05).

Whilst the two stents were not statistically different in terms of stone clearance, nevertheless, the Visiostar grooved stent exhibited a 10% higher SFR (81.2%) compared to the Percuflex stent (70.8%). However, to the urologist and the patient, such differences, although not statistically significant, could nevertheless translate to clinically significant implications. For instance, the Visiostar grooved stent increased the stone-clearance rate to a level closer to that noted among unstented patients (93–95%) [5,24,25], while preventing steinstrasse formation that could be observed among unstented patients, thus resulting in possibly fewer auxiliary procedures (e.g. ureteroscopy).

The presence of a stent per se compromises the intra-ureteric space that remains available around the stent. Hence, generally, compared to unstented patients, stented patients have a lower stone-clearance rate probably because of the less available intra-ureteric space and less urinary flow around the stent. Despite the Visiostar having a larger circumference (7 F) than the Percuflex smooth stent (6 F), the Visiostar’s grooves on the external surface create more intra-ureteric space around the stent that allows extraluminal urinary flow and passage of stone fragments. The importance of such urinary flow around the stent has been noted in animals, where non-smooth (i.e. spiral or grooved) stents created more space around the stent and better extraluminal urinary flow compared to smooth-walled stents [26,27]. This suggests that a smaller diameter Visiostar grooved stent (e.g. 6 F) could possibly further enhance stone clearance.

In terms of stent-related technical challenges, we were unable to insert the Visiostar stent in two patients, although both subsequently had the Percuflex inserted smoothly. We feel that such failure of Visiostar stent insertion for these two patients might be related to the stent’s size (7 F, not suitable for some tight ureters) or the stent’s coating material. The Percuflex Plus is coated by HydroPlus hydrophilic coating that is lubricious and hence causes less urothelial friction and smoother insertion.

As for stent-related complications, the overall complication rates were comparable to previous studies [13]. None of the patients developed stent encrustation or migration probably due to the relative short duration of indwelling the stents (<90 days) [13].

In terms of patient satisfaction, we employed a validated ureteric stent satisfaction questionnaire to assess the impact on health-related quality of life (seven domains) after ureteric stent insertion [22]. The Visiostar exhibited more urinary symptoms compared to the Percuflex (borderline significant) and was associated with significantly more UTIs that were self-reported by the patients through the questionnaire. However, although our hospital data showed that none of the patients had clear cut evidence of UTI or required hospitalisation, these patients reported receiving antibiotics prescribed at other private facilities. Increased UTI may be related to one of two reasons. The first is the probability of having infection prior to stenting [13]. However, comparing the preoperative data of the two groups, the mean preoperative leucocytosis and UTI as indications for stenting were comparable between both groups (*P* = 0.3 and *P* = 0.8, respectively). The second reason may be related to the stent coating. The Percuflex Plus stent is coated by HydroPlus, a hydrophilic coating that supposedly reduces the formation of a conditioning film around the stent and thus reduces the biofilm and consequently decreases bacterial colonisation and infection. However, this hydrophilic coating is still being evaluated for its efficacy [28].

There were no differences between both stents in terms of general satisfaction, or the domains of body pain, general health, work performance, and sexual matters. The more stent-related symptoms and less patient tolerance to the Visiostar stent may be attributed to: i) more UTIs, ii) stent-coating material and hardness of stent body and distal (vesical) pigtail (Percuflex Plus has hydrophilic Hydroplus coating and softer co-polymer material with an even softer pigtail that softens at body temperature) [29], and iii) diameter (Percuflex is of smaller diameter, causing less irritation) [30,31].

### Limitations

The present study has limitations. Traditionally, the ideal way to assess a new device is to conduct a pilot study prior to proceeding to the clinical trial; however, anecdotal evidence from our daily practice showed good stone clearance in patients undergoing placement of Visiostar stents before ESWL. As there was no formal evidence, the team decided to scientifically evaluate the stent via a RCT.

Ideally, the same stent size would have been used for comparison to avoid findings that are attributed to size. We could have compared the Visiostar stent to a 7 F Percuflex; however, we preferred to compare the Visiostar stent to the mostly used 6 F smooth stent to provide real-world clinical evidence rather than theoretical results. To our knowledge,no previous studies or clinical trials in humans addressed such comparisons this. Hence, it was not possible to get guidance in terms of sample size . A larger sample size would have been beneficial, as we observed several border line statistically insignificant results. Computed tomography could be used to confirm stone clearance instead of X-ray and ureteroscopy. However, obtaining a scan might prolong the stent placement, and most patients elected for ureteroscopy, which has the additional benefit of allowing the removal of any possible residual fragments.

Despite this, the present study has strengths. Starting with patient groups similar in their baseline demographic, renal and stone characteristics, it is the first evaluation and RCT to assess a grooved stent in clinical practice. Such findings enhance the non-existent evidence base in terms of comparison of grooved and smooth stents and could be key to the further development of stents in order to enhance stone clearance. Based on the findings of the present study, where ureteric stents are needed and ESWL is planned as a definitive treatment afterwards instead of the more invasive ureteroscopy option, a modified grooved stent would be a better option than the currently commonly used stents. It might be useful for manufacturing companies to produce a grooved stent with smaller outer diameter, softer lower pigtail material and less irritating coating to allow more peri-stent intra-ureteric space that enhances the passage of stone fragments with less stent-related symptoms.

## Conclusions

Although grooved stents did not display a statistically significant effect on the overall SFR and patients’ tolerance when compared to smooth stents, they might nevertheless enhance the stone clearance after lithotripsy, probably by allowing the passage of small stone fragments through the grooves. Compared to smooth stents, patients with grooved stents reported less tolerance as reflected by more UTIs. Further multicentre studies among larger patient populations are required.

## Abbreviations

CONSORT: Consolidated Standards of Reporting TrialsESWL: external shockwave lithotripsySFR, stone-free rateUSSQ: Ureteric Stent Symptoms Questionnaire
